# A unique case of prenatal diagnosis of vascular Ehlers‐Danlos syndrome

**DOI:** 10.1002/ijgo.70431

**Published:** 2025-08-07

**Authors:** Emma Bertucci, Sofia Ceffa, Sartor Giovanna, Maria Gnazzo, Antonio Novelli, Antonio La Marca

**Affiliations:** ^1^ Prenatal Medicine Unit, Obstetrics and Gynecology Unit, Department of Medical and Surgical Sciences for Mother, Child and Adult University of Modena and Reggio Emilia Modena Italy; ^2^ Obstetrics and Gynecology Unit, Department of Medical and Surgical Sciences for Mother, Child and Adult University of Modena and Reggio Emilia Modena Italy; ^3^ Genetic Unit AUSL Modena Italy; ^4^ Laboratory of Medical Genetics, Translational Cytogenomics Research Unit Bambino Gesù Children's Hospital, IRCCS Rome Italy

**Keywords:** *COL3A1*, genetic diagnosis, prenatal diagnosis, vascular Ehlers‐Danlos syndrome

## Abstract

We present a rare instance of prenatal diagnosis of vEDS without a family history. The suspicion of a genetic syndrome arose from an incidental ultrasound finding of a facial anomaly—previously associated with vEDS in adulthood but never described prenatally.

Ehlers‐Danlos syndrome (EDS) encompasses a group of hereditary connective tissue disorders caused by genetic mutations affecting collagen structure and synthesis. Different subtypes involve various types of collagen or related proteins, resulting in diverse clinical features.^1^ The vascular type (vEDS) is the most severe, because of its association with life‐threatening arterial, intestinal, and uterine ruptures.^1^, ^2^ Diagnosis often occurs in childhood, but mild forms may remain unrecognized until adulthood. Prenatal diagnosis is usually performed in families with a known mutation, either through preimplantation genetic diagnosis or invasive prenatal testing. However, ultrasound‐based prenatal diagnosis remains challenging because of the absence of specific markers.^1^, ^3^

We report a rare case of prenatal diagnosis of vEDS in a patient with no family history. The diagnosis followed the detection of a somatic malformation in the second trimester—an unprecedented prenatal finding—associated with a previously undescribed pathogenic variant.

A patient with a history of two miscarriages underwent a normal first‐trimester ultrasound and low‐risk non‐invasive prenatal testing for chromosomal anomalies. She was referred to our center following the early detection of micrognathia and trigonocephaly on anomaly scan.

A detailed anomaly ultrasound performed at 16^+2^ weeks of gestation confirmed the presence of trigonocephaly and micrognathia, with an inferior facial angle of 42°, which falls below the normal range (Figure 1a,b). Trigonocephaly, resulting from the premature fusion of the metopic suture, was identified in the early second trimester, raising concerns about abnormal cranial development. Although it can be an isolated, cosmetic finding, early detection warrants further investigation for potential syndromic or genetic causes, especially when associated with micrognathia, as in this case. Based on the ultrasound, we considered several genetic conditions. Craniosynostosis syndromes like Crouzon or Pfeiffer. We also thought about chromosomal issues like trisomy 13 and 18, along with syndromes such as Kabuki and Bohring‐Opitz that involve facial features. Given the micrognathia, Pierre Robin, Treacher Collins, and CHARGE syndrome were also on the list. We did not rule out connective tissue disorders because of their potential prenatal signs.

**FIGURE 1:**
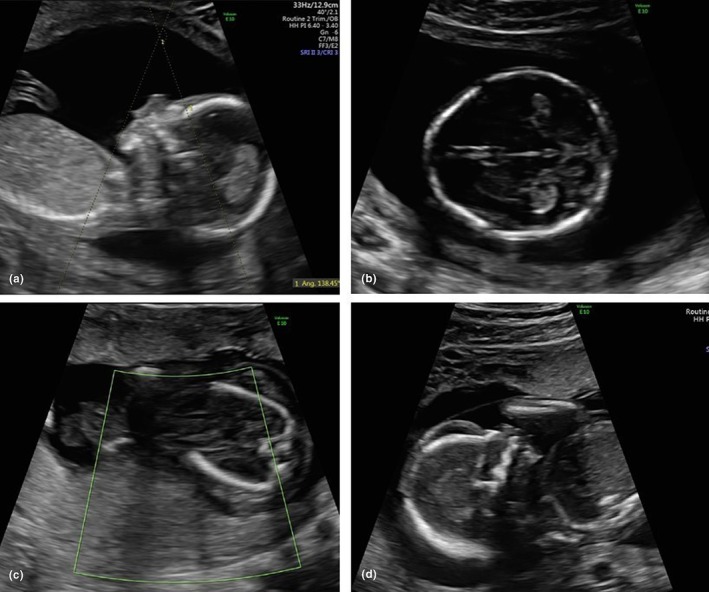
(a, b) Early anomaly scan at 16^+2^ weeks of gestation: facial profile showing micrognathia with an inferior facial angle of 42° and trigonocephaly. (c, d) Ultrasound at 19^+3^ weeks of gestation: absence of cardiac activity on color Doppler and hydrops of the chest and abdomen, generalized hydrops also involving the face.

Amniocentesis was recommended and subsequently performed. Initial analysis revealed a 46XY karyotype, and array‐comparative genomic hybridization (CGH) showed no chromosomal imbalances. Furthermore, karyotyping and array‐CGH testing of both parents yielded normal results. At the follow‐up ultrasound at 19^+3^ weeks, the fetus was found to have no cardiac activity, accompanied by a newly identified finding of generalized hydrops (Figure 1c,d). Labor was subsequently induced, resulting in the delivery of a male fetus. Histologic examination of the fetus did not report any evident somatic malformations, but was limited by advanced tissue autolysis, especially affecting the facial structures; however, overlapping skull bones and fetal edema were observed. While overlapping sutures often result from loss of cranial tone after death, their presence alongside generalized edema suggests a more complex intrauterine pathology. Additionally, histologic examination of the placenta revealed a slightly reduced‐weight placenta, feto‐maternal malperfusion, and superficial implantation.

Given the normal results of both the karyotype and array‐CGH, exome sequencing was proposed using the amniotic fluid obtained through amniocentesis, which identified a de novo heterozygous c.4109A>G (p.Tyr1370Cys) variant in the *COL3A1* gene, associated with vEDS. The test on the parents yielded negative results.

Vascular Ehlers‐Danlos syndrome (vEDS, MIM # 130050) is a rare condition characterized by vascular and tissue fragility, with high mortality due to an alteration in the *COL3A1* gene, which encodes the pro‐α1 chains of type III procollagen, a fibrillar collagen that is found in extensible connective tissues such as skin, lung, uterus, intestine, and the vascular system, frequently in association with type I collagen.^4^, ^5^ Exome sequencing data analysis revealed the heterozygous de novo NM_000090.4:c.4109A>G; NP_000081.2:p.Tyr1370Cys variant in *COL3A1* (MIM * 120180). The COL3A1 missense variant was localized in exon 50 at highly conserved Tyr residue, was absent from gnomAD (v2.1.1), had deleterious in silico predictions (CADD (PHRED) score of 29 and REVEL score of 0.916) and has not been previously reported in the literature. *COL3A1* is highly intolerant to variation (*Z*‐score missense 4.09; pLI 1.00). According to American College of Medical Genetics and Genomics guidelines, the classification of the variant is Likely Pathogenic, class 4 (PS2_strong, PP3_mod, PM2_supp, PP2_supp).

Facial anomalies in vEDS, including micrognathia, a thin vermilion border, a narrow nose, and prominent eyes, have been reported in postnatal life.^4^ The disease often manifests in late adolescence or early adulthood, with complications such as dissections, aneurysms, and arteriovenous fistulae affecting medium‐sized arteries. In pregnant women, vEDS can lead to uterine rupture.^4^, ^5^ Fetal vEDS is primarily associated with preterm birth, low birth weight, and premature rupture of membranes, likely due to reduced type III collagen, which supports amniotic sac integrity.^6^, ^7^ Anomalies such as hip dislocation and amniotic band syndrome are common in fetal vEDS, with amnion rupture thought to result from increased membrane fragility.^5^, ^6^, ^7^

Determining the precise role of genetic alterations in late spontaneous miscarriage is difficult because of limited data. Type III collagen also plays a role in the umbilical cord's structure, which could contribute to its fragility. Maternal‐fetal malperfusion was observed, possibly linked to the *COL3A1* alteration, as compromised collagen integrity increases complication risk. In vEDS, mutations in this gene result in fragile vasculature and impaired tissue integrity, which may predispose to placental abnormalities such as infarction, thrombosis, or poor vascularization. Although not directly reported, murine studies suggest that vEDS may affect placental implantation and vascular development.^8^

In conclusion, this case presents several novel aspects. First, it represents a rare instance of prenatal diagnosis of vEDS without a family history. Second, the suspicion of a genetic syndrome arose from the incidental ultrasound detection of a facial malformation, which has never been previously described in the prenatal period. Third, this case highlights the diagnostic utility of prenatal exome sequencing, especially in situations where imaging findings suggest a genetic condition, but are not specific to a single diagnosis. Fourth, the identified likely pathogenic variant is novel and has not been reported in the literature. Finally, there is a possible association between placental implantation, feto‐maternal malperfusion, and alteration of type III collagen in vEDS fetuses.

## AUTHOR CONTRIBUTIONS

EB acquired the data and contributed to the study design and drafting of the manuscript. EB, SC, SG, MG, and AN critically revised the manuscript for important intellectual content, approved the final version to be published, and agree to be accountable for all aspects of the work. ALM contributed to the study design and drafting of the manuscript and agrees to be accountable for all aspects of the work.

## CONFLICT OF INTEREST STATEMENT

The authors have no conflicts of interest.

## Data Availability

Research data are not shared.
